# Draft Genome Sequences of *Sarcina ventriculi* Strains Isolated from Wild Japanese Macaques in Yakushima Island

**DOI:** 10.1128/genomeA.01694-15

**Published:** 2016-02-04

**Authors:** Kazunari Ushida, Sayaka Tsuchida, Yoshitoshi Ogura, Tetsuya Hayashi, Akiko Sawada, Goro Hanya

**Affiliations:** a Graduate School of Life and Environmental Sciences, Kyoto Prefectural University, Kyoto-shi, Kyoto, Japan; b Department of Bacteriology, Faculty of Medical Sciences, Kyushu University, Fukuoka-shi, Fukuoka, Japan; c Primate Research Institute, Kyoto University, Inuyama-shi, Aichi, Japan

## Abstract

We report the draft genome sequences of *Sarcina ventriculi* strains 14 and 17, both isolated from feces of wild Yakushima macaques (*Macaca fuscata yakui*). These genomic sequences will be helpful for the phylogenetic consideration of the family *Clostridiaceae* and understanding of the contribution of intestinal microbiota to the survival of Yakushima macaques.

## GENOME ANNOUNCEMENT

*Sarcina ventriculi* was first recorded in 1842 in human gastric contents (1) and was demonstrated to have wide distribution with relatively high population density in humans (2). This Gram-positive coccus is well known for its tetrad formation and has been recognized as a causative agent for gastric disorders in both humans and livestock (3, 4).

We isolated two tetrad-forming bacteria, strains 14 and 17, from fresh feces of wild Yakushima macaques (*Macaca fuscata yakui*). They were identified as *S. ventriclui* by partial 16S rRNA gene sequence as indicated elsewhere (5). Illumina Miseq produced 2,278,246 and 3,479,870 pair-ended reads, respectively, for genomes of strains 14 and 17. The reads were assembled by Platanus after trimming of low-quality sequences (6). The number of scaffolds and the total and *N*_50_ lengths of draft genome assemblies were 91 scaffolds (total = 2,474,230 bp, *N*_50_ = 74,523) and 32 scaffolds (total = 2,479,673 bp, *N*_50_ = 260,452), respectively, for strains 14 and 17.

Gene identification and annotation were performed as indicated elsewhere (7). Average nucleotide identities (ANIs) of strains 14 and 17 against a reference clostridial genome (NC_003366) were calculated by ANI calculator (8). As complete assembly of the 16S rRNA genes was not achieved, we obtained the 16S rRNA gene sequences of each strain by mapping Miseq reads to the *S. ventriculi* DSM286 16S rRNA (X76649).

The GC contents of the genomes of strains 14 and 17 (27.20% and 27.18%) were similar to those of the known closest phylogenetic neighbor, *Clostridium perfringens* (28.2% to 28.6%), but the genome sizes were smaller than *C. perfringens* (2.8 to 3.3 Mb) (9, 10). ANIs of the two genomes against *C. perfringens* were 77.75% and 77.64%, respectively, for strains 14 and 17. The full-length sequences of 16S rRNA genes were both 99.5% identical to those of strain DSM286, but intercistronic sequence polymorphism between 16S rRNA gene copies was suggested for both strains.

The draft genomes of strains 14 and 17 each contain 2,312 and 2,311 protein coding sequences (CDSs) and specific clusters of orthologous groups (COGs) were assigned to 1,172 and 1,173 CDSs, respectively. Among these, genes for information processing showed the highest prevalence (26% for both strains) followed by those for carbohydrate metabolism (17% for both strains). Genes encoding the factors for cell division topological specificity (*minEDC* and *maf*) were identified and they may be concerned with tetrad formation of this species (11). Other notable features are the presence of genes for *p*-coumaric acid decarboxylase and cyanate metabolism, both of which may be required for digesting relatively toxic food ingested by monkeys. Genomic sequences of *S. ventriculi* 14 and 17 will be helpful for further phylogenetic consideration of family *Clostridiaceae* and for understanding the contribution of intestinal microbiota to the survival of Yakushima macaques living in the snow-covered mountains of Yakushima Island.

### Nucleotide sequence accession numbers.

The draft genome sequences are available at DDBJ/EMBL/GenBank under the accession numbers BCMV01000001 to BCMV01000091 and BCMW01000001 to BCMW01000032. 16S rRNA gene sequences were deposited as LC101491 and LC101492.
